# Mutations in the *ST7/RAY1/HELG* locus rarely occur in primary colorectal, gastric, and hepatocellular carcinomas

**DOI:** 10.1038/sj.bjc.6600942

**Published:** 2003-06-10

**Authors:** S Yoshimura, T Yamada, S Ohwada, T Koyama, K Hamada, K Tago, I Sakamoto, I Takeyoshi, T Ikeya, F Makita, Y Iino, Y Morishita

**Affiliations:** 1 Second Department of Surgery, Gunma University Faculty of Medicine, 3-39-15, Showa-machi, Maebashi, Gunma 371-8511, Japan; 2 Maebashi Red Cross Hospital, 3-21-36, Asahi-cho, Maebashi, Gunma 371-0014, Japan; 3 National Nishi-Gunma Hospital, 2854, Kanai, Shibukawa, Gunma 377-8511, Japan; 4 Department of Emergency and Critical Care Medicine, Gunma University Faculty of Medicine, 3-39-15, Showa-machi, Maebashi, Gunma 371-8511, Japan

**Keywords:** *ST7*, *RAY1*, *HELG*, 7q31, mutation, tumour-suppressor gene, colorectal cancer, gastric cancer, hepatocellular carcinoma

## Abstract

Human cancers frequently show a loss of heterozygosity on chromosome 7q31, which indicates the existence of broad-range tumour-suppressor gene(s) at this locus. Truncating mutations in the *ST7* gene at this locus are seen frequently in primary colon cancer and breast cancer cell lines. Therefore, the *ST7* gene represents a novel candidate gene for the tumour suppressor at this locus. However, more recent studies have reported that *ST7* mutations are infrequent or absent in primary cancer and cell lines. To ascertain the frequency of mutations of the *ST7* gene in cancer cells, we examined mutations in the *ST7* coding sequence in 48 colorectal, 48 gastric, and 48 hepatocellular carcinomas using polymerase chain reaction–single-strand conformational polymorphism and direct sequencing. We detected somatic mutations, which were located near the exon–intron junction in intron 8, in only three out of 144 cases. We conclude that mutations in the *ST7* gene are rare in primary colorectal, gastric, and hepatocellular carcinomas.

Loss of heterozygosity (LOH) on human chromosome 7q31.1 is found frequently in different human neoplasms, which include cancers of the colon (Zenklusen *et al*, 1995), stomach (Nishizuka *et al*, 1997), pancreas (Achille *et al*, 1996), breast (Bieche *et al*, 1992), prostate (Latil *et al*, 1995), ovary (Edelson *et al*, 1997; Koike *et al*, 1997), head and neck (Zenklusen *et al*, 1995), kidney (Shridhar *et al*, 1997), myeloid system (Liang *et al*, 1998; Koike *et al*, 1999), and thyroid gland (Zhang *et al*, 1998). Previous studies on these cancers have suggested the existence of broad-range tumour-suppressor gene(s) in this chromosomal region.

To date, several genes, such as *CAV1, CAV2* (Chang *et al*, 1994), *MET* (Vande Woude *et al*, 1997), *CAPZ* (Caldwell *et al*, 1989), *WNT2* (Dale *et al*, 1996), *ALP1* (Zenklusen *et al*, 2001), and *CFTR* (Seibert *et al*, 1997), have been located within this region. However, these genes are rarely inactivated by mutations or aberrant promoter methylation. The tumour-suppressor gene(s) responsible for this critical region have not yet been identified (Zenklusen *et al*, 1999).

The *ST7* gene, which in other contexts is designated as *RAY1* (Vincent *et al*, 2000) or *HELG* (Hughes *et al*, 2001), maps within this critical region. Recently, frameshift mutations in the *ST7* gene have been observed frequently in primary colon cancer and breast cancer cell lines (Zenklusen *et al*, 2001). The introduction of *ST7* cDNA suppressed the tumorigenicity of a prostate cancer cell line *in vivo* (Zenklusen *et al*, 2001). These results suggest that the *ST7* gene is a candidate tumour-suppressor gene within this critical region. However, there have been reports that somatic mutation in the *ST7* gene is extremely rare (Hughes *et al*, 2001; Thomas *et al*, 2001; Brown *et al*, 2002; Dong and Sidransky, 2002). Thus, the previous data on *ST7* gene mutations show conflicting results.

In this study, we investigated the true frequency of *ST7* gene mutations by examining 48 primary colorectal cancers, 48 primary gastric cancers that frequently show LOH on 7q31 (Nishizuka *et al*, 1997), and 48 primary hepatocellular carcinomas that show high-level expression of the *ST7/RAY1* gene (Vincent *et al*, 2000; Zenklusen *et al*, 2001). We surveyed mutations in the entire *ST7* coding sequence using polymerase chain reaction–single-strand conformational polymorphism (PCR–SSCP) analysis and direct DNA sequencing.

## MATERIALS AND METHODS

### Tissue specimens and DNA extraction

Specimens from 48 colorectal, 48 gastric, and 48 hepatocellular carcinomas and corresponding noncancerous tissues were obtained at surgery from Japanese patients. The samples were frozen immediately in liquid nitrogen and stored at −80°C until use. High-molecular-weight DNA was extracted using the standard phenol/chloroform procedure.

### Polymerase chain reaction–single-strand conformational polymorphism analysis

All samples were examined by PCR–SSCP analysis for mutations throughout the entire coding sequence of the *ST7* gene (exons 1a–16b). The exon–intron boundaries were identified by comparing the cDNA sequences of *ST7* (GenBank accession no. AY009152) and the genomic DNA sequence of chromosome 7q31 (AC002542). Using this information, we designed intronic primers for each genomic region, except for exons 1b and 16b (Table 1

**Table 1:** Primer sequences for PCR–SSCP analysis of the *ST7*gene

			Primer sequences (5′–3′)	
Exon	Product size (bp)	*T*_m_ (°C)	Sense	Antisense
1a	273	62	gaatcatcccggcagacac	gcgcgagttgcactaacttt
1b	234	58	agcagagaggagcgctgaa	ttgcactaactttccggggc
2	148	62	ccttgttcttctccctttctc	ttaaatgagaaggactccacc
3	230	58	aacagtgaccataaacacgct	aaataatattgcaaactgaagg
4	162	62	gtagtgtcactgaacttacgc	ctgtctttgctctctgaacc
5	369	58	aggtcttgcttttctctctca	gaggggactcatttcaacata
6	220	62	ggattgacttggtgttttctc	atcctccagttcaaatgcagt
7	176	62	gtgactctctctgaatgttcc	tcatttggttagaagtagggc
8	237	62	ggctttgtaattgatggtggc	acaattctgatccccccaatgc
9	342	58	tcaacatcctcactcaaaagc	tctgtaagccactgatcccaa
10	195	58	attccttggtttcttctgccc	gggaaaatacatcaaaagagg
11	191	58	cctgcaaacttatgtgttcct	aacacatctcaattccggtca
12	168	62	ggatggtttttgtctttctgc	atcataacgagttcctgtggg
13	237	62	attaacacaagtgtgtcctgc	ttagcaccttttcatgctctt
14	209	62	cacaaacattggacatctctg	ctggctgaagagaggtgaga
15	351	58	gggtcagatgttggctatgg	cttggctttccccatccatt
16a	200	62	ggtttctgctgacttctgtg	aaggagttggcacagaggag
16b	225	58	aggcgagtgcaatcagaaag	gaggaggagcagttttggtg

). The primers for exons 1b and 16b were prepared as described previously (Thomas *et al*, 2001).

The genomic DNA template (50 ng) was incubated in a total volume of 10 *μ*l PCR buffer that contained 10 mM Tris-HCl (pH 9.0), 50 mM KCl, 1.5 mM MgCl_2_, 100 nM of each primer, 200 *μ*M of each deoxynucleotide triphosphate, 1.5 Ci of alpha-[^32^P]dCTP (Amersham Pharmacia), and 0.5 U of *rTaq* DNA polymerase (TaKaRa). The following PCR conditions were employed: 30 s at 95°C, 30 s at 58°C or 62°C, and 90 s at 72°C for 35 cycles, followed by 10 min at 72°C in a thermal cycler (GeneAmp 9700; Applied Biosystems). Single-strand conformational polymorphism analysis was performed with the low-pH buffer system, which allowed improved separation of fragments of up to 800 bp in length (Kukita *et al*, 1997). The ^32^P-labelled PCR products were denatured, loaded on nondenaturing polyacrylamide gels that contained 10% polyacrylamide (99 : 1 acrylamide to bisacrylamide) and TPE (30 mM Tris (pH 6.8), 20 mM PIPES, and 1 mM Na_2_EDTA), and electrophoresed in TPE buffer at 10°C. The gels were dried and analysed with the BAS 2000 system (Fuji Photo Films). To exclude potential PCR artefacts, all positive cases were tested independently at least three times.

### Sequencing analysis

PCR fragments that showed different mobilities were purified using the QIAquick PCR Purification Kit (QIAGEN), and directly sequenced in both directions using the BigDye Terminator Kit and the ABI 3100 DNA Sequencing System (Applied Biosystems).

### Analysis of microsatellite instability

We assessed microsatellite instability using five reference markers (D2S123, BAT25, BAT26, D5S346, and D17S250) and the criteria recommended by the National Cancer Institute workshop (Boland *et al*, 1998; Yamada *et al*, 2002).

### Statistical analysis

Statistical analysis was performed using the StatView 5.0. program (SAS Institute Inc.). The *χ*^2^, Fisher's exact, and Mann–Whitney *U*-tests were used for background and clinicopathological data. A *P*-value of less than 0.05 was considered to be statistically significant.

### Ethics

This study was carried out with the approval of the ethical committee of Gunma University Faculty of Medicine.

## RESULTS

We detected a somatic mutation in the polypyrimidine tract within the splice-acceptor site of the intron 8–exon 9 junction. Deletions in intron 8 (−32 nucleotides from exon 9) were found in one out of 48 (2.1%) of the colorectal cancers, and in two out of 48 (4.1%) of the gastric cancers (Table 2

**Table 2:** Mutations in the *ST7* gene

Case	Type of tumour	Locations	Mutations	Amino-acid substitution	Status	Microsatellite instability
CRC39	Colorectal	Intron 8 (−32 nt from exon 9)	1 to 3 nt deletion (heterogeneous)	None	Somatic mutation	MSI-H
GC18	Gastric	Intron 8 (−32 nt from exon 9)	1 to 3 nt deletion (heterogeneous)	None	Somatic mutation	MSI-H
GC28	Gastric	Intron 8 (−32 nt from exon 9)	1 to 3 nt deletion (heterogeneous)	None	Somatic mutation	MSI-H
CRC18	Colorectal	Exon 5	427 G to A (heterozygous)	143 Ala to Thr	Germline mutation or rare polymorphism	MSS

nt=nucleotide; CRC=colorectal cancer; GC=gastric cancer; MSI-H=microsatellite instability-high; MSS=microsatellite stable.

). The number of deleted nucleotides in one tumour sample ranged from one to three bases, which demonstrates the highly heterogeneous nature of the tumour. A representative case is shown in Figure 1

**Figure 1:**
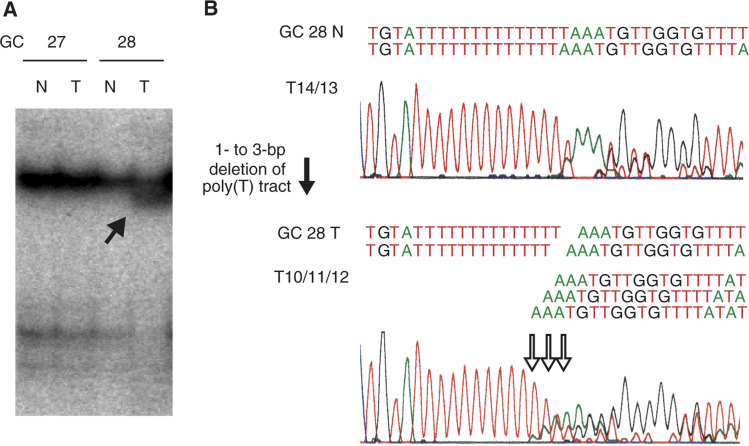
Representative example of an *ST7* frameshift mutation. (**A**) SSCP analysis of the intron 8–exon 9 junction of the *ST7* gene. The solid arrow indicates a shifted band in the tumour sample. T: tumour samples; N: corresponding normal tissue samples. (**B**) Sequence analysis. The open arrow indicates deletions in the polypyrimidine tract within the splice-acceptor site of intron 8 (−3 nucleotides from exon 9). The number of nucleotides deleted ranged from one to three.

. All the three patient groups exhibited high-frequency microsatellite instability (microsatellite instability-high; MSI-H).

**Figure 2:**
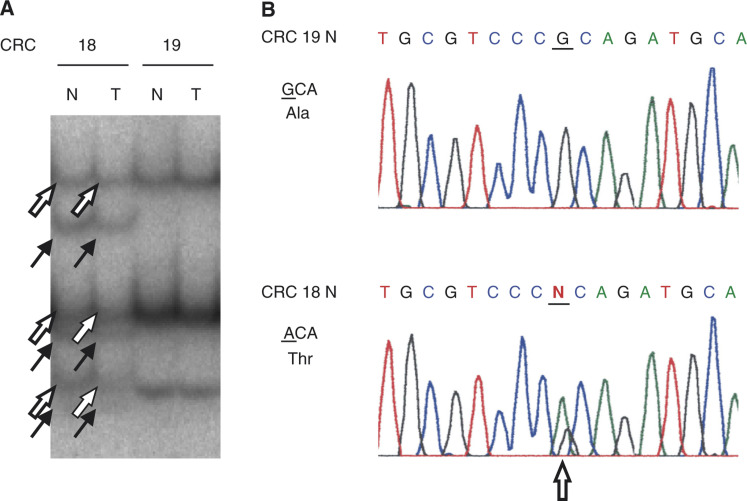
Representative example of a 1-bp substitution in the coding sequence of the *ST7* gene. (**A**) SSCP analysis of the intron 8–exon 9 junction of the *ST7* gene. The solid arrow indicates the shifted band that was predicted to carry substitutions, in both the tumour and corresponding normal tissue sample. The open arrow indicates another allele without a substitution. T: tumour samples; N: corresponding normal tissue samples; CRC=colorectal cancer. (**B**) Sequence analysis. The open arrow indicates a one-nucleotide substitution in exon 9 of the *ST7* gene.

We also detected a G to A substitution at the first nucleotide of codon 143 (GenBank accession no. AY009152) in exon 5 of one of the colorectal cancer cases (Figure 2

, Table 2). This substitution resulted in an amino-acid change from Ala to Thr. The same substitution was found in the corresponding normal tissue from the same patient. Thus, the change represents a germline mutation or rare polymorphism.

In addition, we identified four single-nucleotide polymorphisms (SNPs) in introns 8, 10, 11, and 15 of the *ST7* gene (Table 3

**Table 3:** Polymorphisms in the *ST7* gene locus detected in this study

		Allele frequency		
Location	Nt	CRC	GC	HCC
Intron 8 (−31 nt from exon 9)	A/T	0.05/0.95	0.02/0.98	0.03/0.97
Intron 10 (+8 nt from exon 9)	T/C	0.20/0.80	0.20/0.80	0.18/0.82
Intron 11 (+17 nt from exon 10)	C/T	0.20/0.80	0.20/0.80	0.16/0.84
Intron 15 (+28 nt from exon 14)	T/G	0.00/1.00	0.01/0.99	0.00/1.00

nt=nucleotide; CRC=colorectal cancer; GC=gastric cancer; HCC=hepatocellular carcinoma. Allele frequency was determined in each of the 48 cancers.

). There were no correlations between these SNPs and the clinicopathological data.

## DISCUSSION

We detected somatic mutations in the polypyrimidine tract within the splice-acceptor site of intron 8, although the frequency of mutation was low. The polypyrimidine tract is essential for efficient branch-point utilisation and splice-site recognition, and deletions within this region affect splicing efficiency (Roscigno *et al*, 1993). We were unable to examine whether the mutation at the polypyrimidine tract induced insufficient splicing, because the appropriate RNA samples were not available. Therefore, we could not confirm the involvement of this mutation in carcinogenesis and the progression of colorectal and gastric cancers. However, considering the fact that mutations were found only in the tumour samples and not in the corresponding noncancerous samples, we cannot exclude the possibility that this mutation confers advantages upon these cancer cells under selective pressure.

All of the cases with the polypyrimidine-tract mutation showed high-frequency microsatellite instability. It is known that a simple mononucleotide repeat can act as a mutational target in tumours that show high-frequency MSI (Perucho, 1999; Yamada *et al*, 2002). Therefore, the polypyrimidine tract, in which we found mutations, may be a mutational target in MSI-positive tumours, and mutations therein may be involved in carcinogenesis and the progression of MSI-positive tumours. Microsatellite instability-high is found rarely in hepatocellular carcinomas, and we could not detect any mutations in the polypyrimidine tract in the 48 cases of hepatocellular carcinoma (Saeki *et al*, 2000; Yamamoto *et al*, 2000; Wang *et al*, 2001).

We also detected a single-nucleotide substitution with amino-acid change in one patient with colon cancer. This substitution was identical to that identified previously in the breast cancer cell line MDA-MB435 (Thomas *et al*, 2001). Since the corresponding noncancerous cell line was not available, these investigators could not determine whether the change was somatic or germline specific. In contrast, we found the same substitution in the corresponding normal tissue from the same patient. Therefore, the change is not somatic, but represents a germline mutation or rare polymorphism. Further functional studies are needed to clarify the ramifications of this amino-acid substitution.

In addition, we detected four SNPs in the *ST7* gene locus. There were no correlations between these SNPs and the clinicopathological data. The consequences of these SNPs for colorectal, gastric, and hepatocellular carcinomas are unclear.

Contrary to the result of Zenklusen *et al*, we rarely detected mutations in the *ST7* gene of patients with colorectal, gastric, or hepatocellular carcinoma, a finding that has been corroborated by other groups (Hughes *et al*, 2001; Thomas *et al*, 2001; Brown *et al*, 2002; Dong and Sidransky, 2002). In our study, there were no technical problems in detecting mutations; the frequent detection of SNPs demonstrates the high sensitivity of our procedure. Although the reason for the discrepancy between our results and those of Zenklusen *et al* is unclear, we (and the aforementioned groups) propose the following possible explanations: the use of selected specimens, the presence of PCR artefacts, and the effects of culture passages (Hughes *et al*, 2001; Thomas *et al*, 2001; Brown *et al*, 2002).

We conclude that *ST7* gene mutations are rare in colorectal, gastric, and hepatocellular carcinomas. Our results do not exclude the possibility that the *ST7* gene is inactivated by other molecular mechanisms, such as aberrant hypermethylation or haplo-insufficiency (Merlo *et al*, 1995; Largaespada, 2001). Since there have been no reports on the expression of the *ST7* gene in cancer cells, further studies are needed to understand the role of this gene in carcinogenesis and the progression of these cancers.
